# Assessment of triglyceride-glucose index and its modified indices in adults with type 2 diabetes mellitus and their relationship with dinner timing

**DOI:** 10.1186/s12902-026-02494-6

**Published:** 2026-08-18

**Authors:** Neelam M. Rathod, Suneeta S. Chandorkar

**Affiliations:** Department of Foods and Nutrition, Faculty of Family and Community Sciences, The Maharaja Sayajirao University of Baroda, Vadodara, Gujarat India

**Keywords:** Type 2 diabetes mellitus, Insulin resistance, Hyperinsulinemia, Triglyceride-glucose index, Modified TyG indices, Dinner timing, Chrononutrition, Endocrinology

## Abstract

**Introduction:**

Type 2 diabetes mellitus (T2DM) is associated with various micro- and macrovascular complications that lead to premature morbidity and mortality. Insulin resistance disrupts glucose disposal, elevates hepatic glucose output, and accelerates β-cell dysfunction, thereby increasing cardiometabolic risk. The triglyceride-glucose (TyG) index and its anthropometric derivatives provide simple and cost-effective surrogate measures of insulin resistance. Habitual late dinner timing may impair glucose and lipid metabolism due to circadian variation. However, the relationship between TyG, TyG modified indices, and chrononutrition behaviour such as dinner timing remains limited in adults with T2DM.

**Methodology:**

The cross-sectional study conducted in Vadodara city, India included 441 adults aged 35–60 years with T2DM of less than five years’ duration and not requiring insulin therapy. Participants were categorized as early (< 21:00; *n* = 200) or late (≥ 21:00; *n* = 241)diners. Anthropometric measurements were obtained using standardized protocols, body composition was assessed by bioelectrical impedance analysis, and dietary intake was evaluated using a 3-day 24-hour dietary recall and food frequency questionnaire. Biochemical parameters were obtained from recent medical records (< 3 days). Data were analyzed using SPSS version 24.

**Results:**

Mean age of participants was 49.3 ± 7.1years and 232 (52.6%)were males. The mean values of TyG, TyG-BMI, TyG-WC (waist circumference), TyG-WHr (waist-to-hip ratio), and TyG-WHtr (waist-to-height ratio) were 4.95 ± 0.24, 135.58 ± 23.14, 478.72 ± 62.82, 4.58 ± 0.47, and 2.98 ± 0.39,respectively. TyG, TyG-BMI, TyG-WC, TyG-WHr, and TyG-WHtr increased progressively across quartiles (all *p* < 0.001) from 4.64 ± 0.11 to 5.26 ± 0.12, 106.23 ± 9.43 to 165.29 ± 12.49, 400.86 ± 25.57 to 561.67 ± 31.30, 4.04 ± 0.17 to 5.21 ± 0.27, and 2.51 ± 0.15 to 3.51 ± 0.24, respectively, accompanied by progressively adverse lipemic, glycaemic, and adiposity profiles. TyG index showed the strongest differentiation for biochemical markers triglycerides (F = 229.870), TG/HDL ratio (F = 133.410), fasting blood glucose (F = 91.928), and HbA1c (F = 32.240). The modified indices showed strongest differentiation for their respective anthropometric components: TyG-BMI for BMI (F = 638.905), TyG-WC for WC (F = 457.098), TyG-WHr for WHr (F = 276.633), and TyG-WHtr for WHtr (F = 398.272). Beyond these formula-derived relations, TyG-BMI showed the strongest differentiation for visceral fat percentage (F = 117.639), TyG-WHr for HDL-C (F = 34.643), and TyG-WHtr for muscle percentage (F = 17.395). Participants with late dinner timing had significantly higher index values than early diners (all *p* < 0.001), with moderate effect sizes (Cohen’s d = 0.55–0.77). The largest between-group differences were observed for TyG (Cohen’s d = 0.77), TyG-WHtr (0.76),and TyG-WC (0.75). Multiple linear regression analyses showed that late dinner timing was an independent positive predictor of TyG (β = 0.365), TyG-BMI (β = 0.265), TyG-WC (β = 0.350), TyG-WHr (β = 0.308), and TyG-WHtr (β = 0.342) after adjusting for sex, age, energy intake, and physical activity (all *p* < 0.001), with the models explaining 7–15% of the variability in these indices. Partial (*r* = 0.26–0.37) and part (*r* = 0.25–0.36) correlation showed its independent relation with indices after adjustment. Female sex was significantly negatively related with TyG-WC (β=-0.112) and significantly positively related with TyG-WHtr (β = 0.133). Increasing age was significantly positively related with TyG-WHr (β = 0.090) and TyG-WHtr (β = 0.093). Physical activity showed a weak inverse, although non-significant, relation with anthropometry related TyG indices (TyG-BMI, TyG-WC, and TyG-WHtr).

**Conclusions:**

Habitual late dinner timing was independently related with higher TyG and modified TyG indices.

**Clinical trial number:**

Not applicable.

## Introduction

Type 2 diabetes mellitus (T2DM) is a chronic metabolic condition marked by persistent elevations in blood glucose arising from inadequate insulin secretion, reduced insulin sensitivity, or the coexistence of both abnormalities [1]. Insulin resistance represents a core pathophysiological defect in T2DM, wherein insulin-responsive tissues such as skeletal muscle, adipose tissue, and the liver show impaired responses to circulating insulin. This impaired signaling reduces peripheral glucose disposal, increases hepatic glucose production, and imposes prolonged functional stress on pancreatic β-cells, ultimately leading to progressive β-cell dysfunction and failure [2].

The global burden of T2DM has escalated rapidly, posing a major public health concern worldwide. Projections suggest that by 2030, nearly one in nine adults approximately 643 million individuals will be living with diabetes [3]. The highest absolute numbers are reported in China, India, and the United States of America, which together account for a substantial proportion of the global disease burden [3]. In low- and middle-income countries such as India, the rising prevalence of diabetes parallels rapid urbanization, population aging, and lifestyle transitions which contribute to the development of insulin resistance. Since the early 1990s, India has experienced a marked increase in diabetes-related morbidity and mortality, with current estimates indicating direct healthcare costs of approximately USD 114 per patient annually and nearly 0.6 million diabetes-related deaths each year [4]. Type 2 diabetes mellitus (T2DM) is linked to multiple microvascular and macrovascular complications, including neuropathy, nephropathy, retinopathy, coronary artery disease, peripheral arterial disease, and cerebrovascular disease, which collectively contribute to increased morbidity and premature mortality [5]. Increased susceptibility of insulin resistance is often attributed to the so called “Asian Indian phenotype”, characterized by higher visceral adiposity, excess ectopic fat deposition in the liver and skeletal muscle, greater insulin resistance at comparatively lower body mass indices, and limited β-cell reserve. Together, these metabolic characteristics accelerate the transition from normoglycemia to prediabetes and overt T2DM, as well as to related complications, when compared with White European populations [6]. Direct assessment of insulin resistance using the hyperinsulinemic-euglycemic clamp method remains the gold standard; however, this technique is complex, time-consuming, and is primarily used for research purposes, limiting its use in routine clinical practice [7, 8]. Surrogate insulin resistance marker Homeostasis Model Assessment for Insulin Resistance (HOMA-IR) require fasting insulin measurement, which may be impractical in resource-limited settings [9]. Consequently, simpler, cost-effective markers that capture the metabolic interplay between glucose and lipid metabolism have gained interest. The Triglyceride-Glucose (TyG) index, calculated as ln [fasting triglyceride (mg/dL) × fasting glucose (mg/dL)] / 2, has emerged as a reliable surrogate marker for insulin resistance [10, 11]. The TyG index showed a significant inverse correlation with the hyperinsulinemic-euglycemic clamp (*r*= -0.68, *p*< 0.005) and demonstrated utility compared with HOMA-IR for identifying insulin resistance among healthy adults [10, 11].

Modified TyG indices incorporates anthropometric measures. TyG-BMI multiplies the TyG index with body mass index to reflect general obesity-related insulin resistance, whereas TyG-WC uses waist circumference to capture central adiposity. TyG-WHr and TyG-WHtr integrate waist-to-hip and waist-to-height ratios, respectively, to account for body fat distribution and height-adjusted central obesity [12–14]. Traditional measures such as body mass index (BMI) provide a general estimate of overall obesity but do not reflect regional fat distribution [15]. To overcome these limitations, several anthropometric indices have been developed. Waist-to-hip ratio (WHr) and waist-to-height ratio (WHtr) assess central adiposity by relating waist circumference to hip circumference and height, respectively [16, 17]. These indices provide a more nuanced evaluation of insulin resistance by combining biochemical and anthropometric markers, which is particularly relevant in populations with heterogeneous body composition.

Diet is a major modifiable determinant of fasting glucose and triglyceride levels, directly influencing TyG and its variants. Diets rich in refined carbohydrates, trans fats, and added sugars may elevate both parameters. In India, the majority of the population consumes lower-than-recommended amounts of pulses, legumes, milk, nuts, and vegetables, while intake of cereals and grains exceeds recommendations [18]. This dietary pattern predisposes individuals to elevated fasting glucose and triglyceride levels.

Several investigations have explored associations between dietary patterns and insulin resistance using the triglyceride-glucose (TyG) index as a surrogate marker [19–22]. However, evidence linking TyG or its anthropometric derivatives to chrononutrition-related behaviors, particularly dinner timing, remains scarce. In an Indian population-based study, approximately 32% of total daily energy intake was consumed during the late evening hours (7:00 p.m. to 11:00 p.m.), with the median dinner time occurring at 10:00 p.m. among healthy adults [23]. In South Asian cultural settings, dinner often represents the principal family meal and is routinely consumed late in the evening. Shared meals within households, or commensality, play a central role in social cohesion and interpersonal interaction, further reinforcing late-night eating practices. These patterns may have important public health consequences, as late-evening meals in South Asian households are frequently energy-dense and may disrupt endogenous circadian rhythms governing glucose and lipid metabolism. Circadian misalignment resulting from delayed food intake has been implicated in impaired insulin sensitivity and adverse metabolic outcomes [24].

Given the escalating burden of T2DM in India and growing recognition of meal timing as a determinant of metabolic health, the present study was undertaken to examine the relation between dinner timing and surrogate indices of insulin resistance in Indian adults with T2DM. We hypothesized that later dinner timing is related with higher insulin resistance, as reflected by the TyG index and its modified variants, among Indian adults with type 2 diabetes mellitus.

## Methodology

### Study design

This cross-sectional investigation was conducted in Vadodara, India, over the period 2020–2023. Participants were consecutively enrolled from the outpatient departments of three diabetes clinics. Men and women aged 35–60 years with a confirmed diagnosis of type 2 diabetes mellitus (T2DM) within the preceding five years and not requiring insulin therapy were eligible for inclusion. Individuals with documented diabetes-related complications or irregular clinic follow-up were excluded. Informed consent to participate was obtained from all participants before enrolment in the study.

A total of 441 individuals fulfilled the eligibility criteria and were included in the analysis. The minimum sample size was estimated at 205 using the formula n = (3.84 × p × q) / L², assuming a 95% confidence level and 5% absolute precision. Prevalence estimates were derived from the National Family Health Survey (NFHS-5) district factsheet, which reported diabetes prevalence of 15.1% in men and 16.6% in women; an average prevalence (p) of 15.9% was therefore used, with q representing (1 - p).

Among the study participants, 200 routinely consumed dinner before 9:00 PM (early dinner), while 241 habitually consumed dinner at or after 9:00 PM (late dinner), with these patterns maintained consistently for more than one year. These groups were compared to evaluate differences in the TyG index and modified TyG indices according to dinner timing. The 9:00 PM (21:00 h) cutoff was selected based on previous literature identifying food intake after 21:00 h as late-evening eating and showing its association with adverse metabolic outcomes. In a nationally representative NHANES analysis of 34,470 adults, Farsijani et al. defined late-night eating as food intake between 21:00 and 23:59 h [25]. Similarly, Crispim et al., in a population-based study of 21,020 Brazilian adults, reported that energy intake after 21:00 h was associated with higher BMI [26]. Experimental studies have shown that consuming dinner at 21:00 h, 22:00 h, and 22.30 h, compared with earlier hours, results in higher postprandial glucose concentrations and increased 24-hour glycaemic variability [27–30]. Therefore, a 21:00 h threshold was considered appropriate for defining dinner timing in the present study. 

### Assessments

Body weight, visceral fat% and body muscle% were measured using a calibrated body composition monitor (BodySCAN™ HBF-224; Omron Healthcare, Kyoto, Japan), with participants wearing light clothing and no footwear. Body height was measured using a portable stadiometer (measurement range 20–210 cm; precision 1 mm). Waist and hip circumferences were measured using a non-stretchable measuring tape according to standard World Health Organization (WHO) protocols. All measurements were recorded using standardized procedures. BMI was calculated as body weight (kg) divided by height squared (m²).

Dietary intake was assessed using a 3-day 24-hour dietary recall, including two weekdays and one weekend day, along with a food frequency questionnaire (FFQ) to assess habitual food consumption patterns. Trained interviewers collected dietary data using standardized household measures. Reported foods were converted into raw ingredient equivalents and analysed using DietCal software (Profound Tech Solutions, New Delhi, India) to estimate macro- and micronutrient intakes. Physical activity was quantified using MET-minutes per week, calculated as METs × duration × days per week.

Biochemical data were retrieved from participants’ latest (within 3 days) medical records. Glycaemic parameters, including fasting blood glucose, postprandial blood glucose, and glycated/glycosylated haemoglobin (HbA1c), along with lipid profile parameters, including total cholesterol, triglycerides, high-density lipoprotein cholesterol (HDL-C), and low-density lipoprotein cholesterol (LDL-C), were recorded. The triglyceride-to-HDL cholesterol ratio (TG/HDL-C) was calculated by dividing fasting serum triglyceride concentration by fasting HDL-C concentration, with both parameters expressed in mg/dL.

### TyG index and modified TyG indices

The TyG index was calculated using fasting blood glucose and fasting triglyceride concentrations according to the following formula:

$$TyG = ln\left[ \matrix{ fasting \ {\rm{ }}triglycerides{\rm{ }}\left( {mg/dL} \right) \hfill \cr \times fasting \ {\rm{ }}{\rm{ }}glucose{\rm{ }}\left( {mg/dL} \right) \hfill \cr} \right]/2$$

where *ln* represents the natural logarithm. The calculation involves multiplying fasting triglyceride and fasting glucose values, applying the natural logarithm to the resulting product, and dividing the obtained value by two. Original developers of the formula have published corrected formula in 2020 [31]. An alternative formula has also been reported in the literature, in which the product of fasting triglycerides and fasting glucose is first divided by two, followed by calculation of the natural logarithm:

These two equations are mathematically different and generate different numerical ranges. For example, for a participant with fasting triglycerides of 186 mg/dL and fasting glucose of 139 mg/dL:

Corrected formula TyG = *ln* (186 × 139) / 2 = ln (25,854) / 2 = 10.160 / 2 = 5.08.

Alternative formula TyG = *ln* [(186 × 139) / 2] = ln (12,927) = 9.47.

Although both formulas have been used in previous studies and have shown the usefulness of the TyG index as a surrogate marker of insulin resistance [31], they produce different absolute values. Therefore, the corrected formula recommended by the original developers was used consistently for all TyG index calculations in the present study.

The TyG-BMI index was obtained by multiplying the TyG index with body mass index (BMI, kg/m²), reflecting general adiposity. The TyG-WC index was calculated as the product of the TyG index and waist circumference (WC, cm), capturing central obesity. Additionally, body fat distribution was assessed using TyG-WHr and TyG-WHtr, computed by multiplying the TyG index by waist-to-hip ratio (WHr) and waist-to-height ratio (WHtr), respectively. These composite indices integrate metabolic and anthropometric parameters and are particularly useful in populations with heterogeneous body composition, such as Asian Indians, where insulin resistance may occur at lower BMI thresholds.

### Chrononutrition assessment

Chrononutrition behaviours were assessed using a pre-tested, structured, interviewer-administered questionnaire, in which the interviewer obtained participants’ recall of their usual meal timings, daily eating window duration, and overnight fasting interval and recorded the responses.

### Statistical analysis

TyG index and modified TyG indices (TyG-BMI, TyG-WC, TyG-WHr, and TyG-WHtr) were calculated using Microsoft Excel. Statistical analyses were performed using SPSS version 24.0 (IBM Corp., Armonk, NY, USA).

Mean ± standard deviation (S.D.), minimum, maximum, and quartile cut points were reported for the TyG and modified TyG indices. Differences in age, anthropometric measurements, body composition, and biochemical characteristics across quartiles of TyG and TyG modified indices were evaluated using one-way analysis of variance (ANOVA). Independent samples t-tests were used to compare the mean values of TyG and its anthropometric variants between dinner timing groups. Effect sizes were calculated using Cohen’s d to quantify the magnitude of between-group differences. Cohen’s d effect sizes (0.2, 0.5, 0.8) represents small effect, medium effect, and large difference, respectively. Multiple linear regression analyses were conducted to examine the independent relation of dinner timing with TyG and its anthropometric variants after adjusting for potential confounders, including sex, age, energy intake, and physical activity. Regression assumptions were assessed using standardized residuals to identify potential outliers and tolerance and variance inflation factor (VIF) values to evaluate multicollinearity. Model fit was assessed using the coefficient of determination (R²), adjusted R², analysis of variance (F-test), and the standard error of the estimate. Regression coefficients are presented as unstandardized coefficients (B), standardized coefficients (β), t-values, and p-values. Zero-order, partial, and part correlations were also reported. For all statistical tests, a two-tailed p-value < 0.05 was considered statistically significant.

## Results

### Distribution and descriptive characteristics of TyG and modified TyG indices among adults with type 2 diabetes mellitus

Among the 441 adults with T2DM, 232 (52.6%) were male and 209 (47.4%) were female. The mean age of male and female participants were 49.8 ± 7.0 years and 48.67 ± 7.28 years, respectively, with an overall age range of 35–60 years. Table 1 reports the mean ± standard deviation (S.D.), minimum, maximum, and quartile cut points of TyG and modified TyG indices of the study population (*n* = 441). The mean TyG value was 4.95 ± 0.24. The corresponding mean values for TyG-BMI, TyG-WC, TyG-WHr, and TyG-WHtr were 135.58 ± 23.14, 478.72 ± 62.82, 4.58 ± 0.47, and 2.98 ± 0.39, respectively.

**Table 1:** Distribution and descriptive characteristics of TyG and modified TyG indices among study participants (*n* = 441)

	TyG	TyG-BMI	TyG-WC	TyG-WHr	TyG-WHtr
Minimum	4.18	84.80	322.09	3.51	2.18
Maximum	5.57	205.27	666.51	6.11	4.12
Quartile 1	4.78	119.15	435.46	4.27	2.70
Quartile 2	4.97	135.68	474.42	4.51	2.95
Quartile 3	5.10	150.58	518.78	4.89	3.23
Mean ± S.D.	4.95 ± 0.24	135.58 ± 23.14	478.72 ± 62.82	4.58 ± 0.47	2.98 ± 0.39

### Distribution of age, anthropometric measurements, body composition, and biochemical parameters according to TyG index and modified TyG indices quartiles among adults with type 2 diabetes mellitus

As shown in Tables 2, 3, 4, 5 and 6, the quartile-wise analysis of TyG and TyG-related indices (TyG-BMI, TyG-WC, TyG-WHr, and TyG-WHtr) showed significant differences in anthropometric measurements, body composition, and biochemical parameters among participants. Mean TyG increased from 4.64 ± 0.11 in the lowest quartile to 5.26 ± 0.12 in the highest quartile (F = 992.98, *p* < 0.001). Similarly, TyG-BMI increased from 106.23 ± 9.43 to 165.29 ± 12.49 (F = 968.32, *p* < 0.001), TyG-WC increased from 400.86 ± 25.57 to 561.67 ± 31.30 (F = 1047.26, *p* < 0.001), TyG-WHr increased from 4.04 ± 0.17 to 5.21 ± 0.27 (F = 890.997, *p* < 0.001), and TyG-WHtr increased from 2.51 ± 0.15 to 3.51 ± 0.24 (F = 850.22, *p* < 0.001) across the lowest to highest quartiles.

Higher quartiles of TyG and modified TyG indices were related with significantly greater adverse lipemic and glycaemic parameters, as well as increased general and central adiposity measures. The original TyG index showed the strongest differentiation for independent biochemical markers, including triglycerides (F = 229.870), TG/HDL ratio (F = 133.410), fasting blood glucose (F = 91.928), and HbA1c (F = 32.240). As expected from their construction, the modified indices showed strong differentiation for their respective anthropometric components: TyG-BMI for BMI (F = 638.905), TyG-WC for WC (F = 457.098), TyG-WHr for WHr (F = 276.633), and TyG-WHtr for WHtr (F = 398.272). Beyond these formula-derived relations, TyG-BMI showed the strongest differentiation for visceral fat percentage (F = 117.639), TyG-WHr for HDL-C (F = 34.643), and TyG-WHtr for muscle percentage (F = 17.395).

**Table 2:** Distribution of age, anthropometric measurements, body composition, and biochemical parameters according to TyG index quartiles among individuals with type 2 diabetes mellitus

Parameters	Quartile 1	Quartile 2	Quartile 3	Quartile 4	F value	*p*-value
**Number of participants**	110	110	111	110		
**TyG**	4.64 ± 0.11	4.88 ± 0.06	5.03 ± 0.04	5.26 ± 0.12	992.98	< 0.001
**Age (years)**	47.92 ± 6.47	50.05 ± 7.66	49.77 ± 7.17	49.34 ± 7.28	1.924	0.125
**Anthropometry**						
Weight (kg)	67.81 ± 13.17	68.47 ± 14.23	73.19 ± 13.77	73.29 ± 13.62	5.133	0.002
Height (cm)	160.88 ± 8.32	160.60 ± 9.67	160.02 ± 8.95	160.34 ± 9.88	0.175	0.913
BMI (kg/m²)	26.12 ± 4.07	26.44 ± 4.42	28.44 ± 3.98	28.33 ± 3.68	10.068	< 0.001
Waist circumference (cm)	93.04 ± 8.65	94.21 ± 10.13	98.96 ± 10.42	99.57 ± 10.88	11.882	< 0.001
Hip circumference (cm)	103.46 ± 6.93	103.66 ± 8.37	105.67 ± 8.46	104.35 ± 8.86	1.635	0.180
Waist hip ratio	0.90 ± 0.06	0.91 ± 0.06	0.94 ± 0.07	0.95 ± 0.07	16.677	< 0.001
Waist height ratio	0.58 ± 0.05	0.59 ± 0.07	0.62 ± 0.07	0.62 ± 0.06	12.892	< 0.001
**Body composition**						
Visceral fat (%)	10.37 ± 3.60	11.07 ± 4.27	13.69 ± 4.23	14.00 ± 4.17	22.181	< 0.001
Muscle (%)	27.59 ± 3.30	26.65 ± 4.58	26.14 ± 3.47	26.05 ± 4.23	3.547	0.015
**Biochemical parameters**						
Total cholesterol (mg/dL)	138.54 ± 25.55	160.55 ± 45.43	183.18 ± 43.82	196.66 ± 42.16	44.919	< 0.001
LDL-C (mg/dL)	104.18 ± 25.13	117.29 ± 28.63	129.85 ± 27.01	135.33 ± 18.94	33.431	< 0.001
HDL-C (mg/dL)	46.43 ± 8.32	45.35 ± 8.52	40.44 ± 7.46	39.30 ± 7.72	21.422	< 0.001
Triglycerides (mg/dL)	96.26 ± 22.06	129.56 ± 28.11	166.05 ± 22.19	210.96 ± 53.68	229.87	< 0.001
TG/HDL ratio	2.18 ± 0.81	2.97 ± 0.90	4.26 ± 1.02	5.68 ± 2.29	133.41	< 0.001
Fasting blood glucose (mg/dL)	116.31 ± 26.62	139.11 ± 29.34	143.95 ± 20.38	184.36 ± 43.09	91.928	< 0.001
Postprandial blood glucose (mg/dL)	167.23 ± 42.30	177.50 ± 44.19	192.94 ± 39.97	234.76 ± 61.66	42.524	< 0.001
HbA1c (%)	6.98 ± 0.78	7.18 ± 0.90	7.68 ± 1.31	8.44 ± 1.63	32.240	< 0.001

**Table 3:** Distribution of age, anthropometric measurements, body composition, and biochemical parameters according to TyG-BMI quartiles among individuals with type 2 diabetes mellitus

Parameters	Quartile 1	Quartile 2	Quartile 3	Quartile 4	F value	*p*-value
**Number of participants**	110	110	111	110		
**TyG-BMI**	106.23 ± 9.43	127.73 ± 4.27	143.00 ± 4.40	165.29 ± 12.49	968.317	< 0.001
**Age (years)**	49.36 ± 6.72	49.27 ± 7.28	49.92 ± 7.37	48.52 ± 7.37	0.709	0.547
**Anthropometry**						
Weight (kg)	56.11 ± 8.41	66.31 ± 7.64	75.80 ± 8.59	84.51 ± 11.07	202.433	< 0.001
Height (cm)	158.68 ± 10.56	159.86 ± 8.71	162.12 ± 8.02	161.15 ± 9.07	2.978	0.031
BMI (kg/m²)	22.22 ± 1.91	25.88 ± 1.12	28.77 ± 1.55	32.46 ± 2.39	638.905	< 0.001
Waist circumference (cm)	85.64 ± 7.22	94.17 ± 6.48	99.04 ± 6.20	106.93 ± 8.04	177.622	< 0.001
Hip circumference (cm)	97.26 ± 6.40	102.43 ± 6.95	106.24 ± 5.53	111.20 ± 6.81	92.229	< 0.001
Waist hip ratio	0.88 ± 0.05	0.92 ± 0.05	0.93 ± 0.07	0.96 ± 0.07	33.609	< 0.001
Waist height ratio	0.54 ± 0.04	0.59 ± 0.05	0.61 ± 0.04	0.67 ± 0.06	126.563	< 0.001
**Body composition**						
Visceral fat (%)	8.31 ± 3.68	11.32 ± 2.34	13.16 ± 2.17	16.35 ± 4.33	117.639	< 0.001
Muscle (%)	28.15 ± 4.24	26.20 ± 4.10	27.03 ± 3.39	25.05 ± 3.44	12.991	< 0.001
**Biochemical parameters**						
Total cholesterol (mg/dL)	148.06 ± 33.37	172.02 ± 50.29	176.61 ± 48.54	182.30 ± 41.31	12.944	< 0.001
LDL-C (mg/dL)	110.67 ± 28.67	122.47 ± 26.28	122.64 ± 29.56	130.93 ± 22.95	10.479	< 0.001
HDL-C (mg/dL)	47.70 ± 8.45	41.50 ± 7.50	42.71 ± 8.63	39.59 ± 7.52	20.393	< 0.001
Triglycerides (mg/dL)	117.72 ± 35.97	146.71 ± 43.37	154.04 ± 55.12	184.47 ± 59.04	34.093	< 0.001
TG/HDL ratio	2.56 ± 0.99	3.70 ± 1.39	3.88 ± 1.86	4.95 ± 2.36	35.232	< 0.001
Fasting blood glucose (mg/dL)	130.55 ± 31.96	142.88 ± 31.45	154.73 ± 46.64	155.47 ± 40.59	10.426	< 0.001
Postprandial blood glucose (mg/dL)	172.95 ± 50.92	188.72 ± 40.99	205.76 ± 57.79	204.88 ± 58.76	9.619	< 0.001
HbA1c (%)	7.04 ± 0.85	7.56 ± 1.29	7.73 ± 1.34	7.95 ± 1.56	10.047	< 0.001

**Table 4:** Distribution of age, anthropometric measurements, body composition, and biochemical parameters according to TyG-waist circumference (TyG-WC) quartiles among individuals with type 2 diabetes mellitus

Parameters	Quartile 1	Quartile 2	Quartile 3	Quartile 4	F value	*p*-value
**Number of participants**	110	110	111	110		
**TyG-WC**	400.86 ± 25.57	454.08 ± 12.14	496.20 ± 12.76	561.67 ± 31.30	1047.255	< 0.001
**Age (years)**	48.37 ± 7.31	48.86 ± 7.09	50.86 ± 6.53	48.97 ± 7.61	2.584	0.053
**Anthropometry**						
Weight (kg)	57.65 ± 9.06	67.97 ± 10.60	74.59 ± 10.41	82.54 ± 11.79	110.582	< 0.001
Height (cm)	157.62 ± 9.57	159.38 ± 8.52	162.05 ± 8.41	162.78 ± 9.42	7.773	< 0.001
BMI (kg/m²)	23.17 ± 2.90	26.69 ± 3.03	28.33 ± 2.64	31.13 ± 3.47	132.850	< 0.001
Waist circumference (cm)	83.89 ± 5.73	93.51 ± 4.42	99.53 ± 3.84	108.84 ± 6.22	457.098	< 0.001
Hip circumference (cm)	97.04 ± 7.08	103.43 ± 6.21	106.24 ± 5.80	110.43 ± 7.32	79.194	< 0.001
Waist hip ratio	0.87 ± 0.05	0.91 ± 0.05	0.94 ± 0.05	0.99 ± 0.06	108.019	< 0.001
Waist height ratio	0.53 ± 0.04	0.59 ± 0.03	0.62 ± 0.04	0.67 ± 0.06	198.069	< 0.001
**Body composition**						
Visceral fat (%)	9.06 ± 3.60	11.18 ± 3.20	12.78 ± 2.20	16.12 ± 4.68	77.988	< 0.001
Muscle (%)	27.59 ± 4.25	27.08 ± 3.68	26.07 ± 3.94	25.70 ± 3.71	5.552	0.001
**Biochemical parameters**						
Total cholesterol (mg/dL)	152.31 ± 35.84	158.84 ± 43.71	173.04 ± 48.81	194.85 ± 41.73	21.307	< 0.001
LDL-C (mg/dL)	111.42 ± 26.94	118.89 ± 26.78	122.08 ± 30.83	134.33 ± 21.30	14.058	< 0.001
HDL-C (mg/dL)	47.33 ± 8.89	44.79 ± 7.75	41.36 ± 7.88	38.04 ± 6.65	29.472	< 0.001
Triglycerides (mg/dL)	117.97 ± 36.94	132.38 ± 42.88	155.88 ± 43.88	196.69 ± 57.23	61.954	< 0.001
TG/HDL ratio	2.62 ± 1.20	3.12 ± 1.39	3.95 ± 1.43	5.39 ± 2.24	61.935	< 0.001
Fasting blood glucose (mg/dL)	130.87 ± 32.54	137.17 ± 32.64	149.15 ± 37.65	166.48 ± 44.45	19.572	< 0.001
Postprandial blood glucose (mg/dL)	176.35 ± 46.25	181.61 ± 45.66	196.72 ± 44.90	217.71 ± 67.30	14.054	< 0.001
HbA1c (%)	7.18 ± 0.99	7.26 ± 0.90	7.50 ± 1.11	8.34 ± 1.79	19.680	< 0.001

**Table 5:** Distribution of age, anthropometric measurements, body composition, and biochemical parameters according to TyG-waist height ratio (TyG-WHr) quartiles among individuals with type 2 diabetes mellitus

Parameters	Quartile 1	Quartile 2	Quartile 3	Quartile 4	F value	*p*-value
**Number of participants**	110	110	111	110		
**TyG-WHr**	4.04 ± 0.17	4.38 ± 0.08	4.70 ± 0.11	5.21 ± 0.27	890.997	< 0.001
**Age (years)**	48.36 ± 7.30	49.43 ± 7.32	49.47 ± 6.48	49.82 ± 7.61	0.842	0.471
**Anthropometry**						
Weight (kg)	63.44 ± 12.05	69.18 ± 14.21	73.68 ± 13.28	76.45 ± 12.50	20.925	< 0.001
Height (cm)	159.53 ± 8.68	160.21 ± 9.35	161.56 ± 9.48	160.53 ± 9.26	0.933	0.425
BMI (kg/m²)	24.89 ± 4.05	26.74 ± 3.71	28.10 ± 3.65	29.60 ± 3.79	30.580	< 0.001
Waist circumference (cm)	87.81 ± 7.79	93.94 ± 8.41	99.41 ± 8.15	104.61 ± 9.06	81.983	< 0.001
Hip circumference (cm)	102.85 ± 7.85	104.09 ± 8.50	106.23 ± 8.28	103.97 ± 7.91	3.322	0.020
Waist hip ratio	0.85 ± 0.04	0.90 ± 0.03	0.94 ± 0.04	1.01 ± 0.05	276.633	< 0.001
Waist height ratio	0.55 ± 0.05	0.59 ± 0.05	0.62 ± 0.05	0.65 ± 0.06	71.816	< 0.001
**Body composition**						
Visceral fat (%)	9.74 ± 3.59	11.06 ± 3.45	13.12 ± 3.55	15.22 ± 4.67	42.855	< 0.001
Muscle (%)	27.95 ± 3.68	26.51 ± 3.65	26.17 ± 3.96	25.80 ± 4.24	6.404	< 0.001
**Biochemical parameters**						
Total cholesterol (mg/dL)	148.79 ± 36.64	160.95 ± 46.22	176.92 ± 47.33	192.34 ± 39.83	21.648	< 0.001
LDL-C (mg/dL)	110.23 ± 27.39	117.28 ± 28.51	127.28 ± 29.54	131.87 ± 19.97	14.787	< 0.001
HDL-C (mg/dL)	47.99 ± 8.03	44.80 ± 8.16	40.31 ± 7.70	38.43 ± 6.91	34.643	< 0.001
Triglycerides (mg/dL)	107.80 ± 28.85	131.62 ± 38.25	167.67 ± 47.72	195.73 ± 53.34	89.385	< 0.001
TG/HDL ratio	2.32 ± 0.80	3.08 ± 1.20	4.37 ± 1.61	5.33 ± 2.19	83.355	< 0.001
Fasting blood glucose (mg/dL)	127.91 ± 32.09	135.96 ± 30.49	146.96 ± 31.33	172.86 ± 46.50	33.087	< 0.001
Postprandial blood glucose (mg/dL)	175.94 ± 43.65	179.87 ± 43.13	196.46 ± 47.07	220.12 ± 67.86	16.785	< 0.001
HbA1c (%)	7.19 ± 0.89	7.19 ± 1.02	7.54 ± 1.10	8.37 ± 1.75	22.390	< 0.001

**Table 6:** Distribution of age, anthropometric measurements, body composition, and biochemical parameters according to TyG-waist height ratio (TyG-WHtr) quartiles among individuals with type 2 diabetes mellitus

Parameters	Quartile 1	Quartile 2	Quartile 3	Quartile 4	F value	*p*-value
**Number of participants**	110	110	110	111		
**TyG-WHtr**	2.51 ± 0.15	2.85 ± 0.07	3.07 ± 0.08	3.51 ± 0.24	850.221	< 0.001
**Age (years)**	48.72 ± 7.14	48.03 ± 6.79	50.19 ± 7.23	50.14 ± 7.41	2.478	0.061
**Anthropometry**						
Weight (kg)	61.30 ± 12.01	70.71 ± 12.44	72.68 ± 12.64	78.02 ± 13.09	34.075	< 0.001
Height (cm)	161.90 ± 10.38	160.73 ± 8.30	161.14 ± 8.60	158.08 ± 9.04	3.677	0.012
BMI (kg/m²)	23.24 ± 3.04	27.19 ± 3.09	27.79 ± 2.87	31.08 ± 3.40	117.960	< 0.001
Waist circumference (cm)	85.19 ± 7.16	94.69 ± 5.60	98.01 ± 6.39	107.80 ± 7.05	222.134	< 0.001
Hip circumference (cm)	98.02 ± 6.57	104.48 ± 6.96	104.67 ± 7.25	109.93 ± 7.41	52.727	< 0.001
Waist hip ratio	0.87 ± 0.04	0.91 ± 0.05	0.94 ± 0.06	0.94 ± 0.07	85.492	< 0.001
Waist height ratio	0.53 ± 0.03	0.59 ± 0.02	0.61 ± 0.03	0.68 ± 0.05	398.272	< 0.001
**Body composition**						
Visceral fat (%)	9.56 ± 3.80	11.02 ± 3.13	12.92 ± 2.99	15.61 ± 4.76	53.980	< 0.001
Muscle (%)	28.28 ± 4.10	27.16 ± 3.58	26.26 ± 3.78	24.75 ± 3.54	17.395	< 0.001
**Biochemical parameters**						
Total cholesterol (mg/dL)	152.40 ± 40.2	153.92 ± 40.9	185.81 ± 48.7	186.77 ± 40.2	22.185	< 0.001
LDL-C (mg/dL)	112.02 ± 26.76	117.37 ± 28.27	126.37 ± 28.87	130.88 ± 23.57	11.088	< 0.001
HDL-C (mg/dL)	47.37 ± 8.13	44.40 ± 8.60	41.35 ± 8.55	38.43 ± 6.05	26.314	< 0.001
Triglycerides (mg/dL)	117.79 ± 36.52	128.83 ± 38.46	167.13 ± 49.54	188.87 ± 58.08	55.986	< 0.001
TG/HDL ratio	2.58 ± 1.02	3.08 ± 1.33	4.30 ± 1.83	5.11 ± 2.16	53.880	< 0.001
Fasting blood glucose (mg/dL)	129.34 ± 31.05	132.18 ± 29.79	157.26 ± 39.30	164.76 ± 43.59	26.294	< 0.001
Postprandial blood glucose (mg/dL)	176.05 ± 48.16	177.87 ± 39.15	204.51 ± 54.76	213.81 ± 62.01	14.870	< 0.001
HbA1c (%)	7.10 ± 0.80	7.29 ± 1.06	7.75 ± 1.40	8.13 ± 1.64	14.746	< 0.001

### Comparison of TyG index and modified TyG indices according to dinner timing among adults with type 2 diabetes mellitus

Table 7 compares TyG and its modified TyG indices between participants consuming dinner early (< 21:00) and late (≥ 21:00). Participants with late dinner timing showed significantly higher values for all indices compared with those consuming dinner earlier. Mean TyG increased from 4.85 ± 0.23 in the early dinner group to 5.03 ± 0.22 in the late dinner group (t = -8.08, *p* < 0.001). Similarly, TyG-BMI (128.83 ± 21.05 vs. 141.18 ± 23.33), TyG-WC (454.40 ± 54.06 vs. 498.03 ± 62.81), TyG-WHr (4.43 ± 0.43 vs. 4.71 ± 0.46), and TyG-WHtr (2.84 ± 0.32 vs. 3.11 ± 0.40) were all significantly higher among late dinner consumers (all *p* < 0.001). Effect sizes were moderate (Cohen’s d = 0.55–0.77), with the strongest differences observed for TyG (d = 0.77), TyG-WHtr (d = 0.76), and TyG-WC (d = 0.75).

**Table 7:** Comparison of TyG index and modified TyG indices according to dinner timing among adults with type 2 diabetes mellitus

Index	Dinner timing	Mean ± S.D.	t-test for independent samples
TyG	Early	4.85 ± 0.23	t(410.9) = -8.08, p = <0.001, 95% CI [-0.22, -0.13] Cohen’s d = 0.77
	Late	5.03 ± 0.22	
TyG-BMI	Early	128.83 ± 21.05	t(439) = -5.79, p = <0.001, 95% CI [-16.56, -8.16], Cohen’s d = 0.55
	Late	141.18 ± 23.33	
TyG-WC	Early	454.40 ± 54.06	t(438.4) = -7.84, p = <0.001, 95% CI [-54.56, -32.69], Cohen’s d = 0.75
	Late	498.03 ± 62.81	
TyG-WHr	Early	4.43 ± 0.43	t(439) = -6.69, p = <0.001, 95% CI [-0.37, -0.20], Cohen’s d = 0.64
	Late	4.71 ± 0.46	
TyG-WHtr	Early	2.84 ± 0.32	t(438.5) = -7.92, p = <0.001, 95% CI [-0.34, -0.20] Cohen’s d = 0.76
	Late	3.11 ± 0.40	

Footnotes: In present study,early dinner was defined as dinner consumed before 21:00 h (< 21:00), and late dinner was defined as dinner consumed at or after 21:00 h (≥ 21:00)

### Independent relation of late dinner timing with TyG and modified TyG indices in adults with type 2 diabetes mellitus

Total energy intake and physical activity levels were comparable among participants. Mean energy intake was  1338 ± 88 kcal/day (range: 1079–1542 kcal/day), while physical activity was 960.48 ± 260.61 MET-min/week (range: 495–1485 MET-min/week). Independent-samples t-tests showed no significant differences between early and late dinner consumers for energy intake (t(439) = 0.54, *p* = 0.59, Cohen’s d = 0.05) or physical activity (t(439) = 1.39, *p* = 0.17, Cohen’s d = 0.13). These findings indicate comparable lifestyle factors across dinner timing groups. Table 8 shows the results of multiple linear regression analyses examining the relation of dinner timing with TyG and modified TyG indices after adjustment for energy intake and physical activity. Dinner timing emerged as a significant independent predictor of the TyG index and all modified TyG indices. Regression diagnostics showed no multicollinearity(VIF 1.01–1.32)and no influential residual outliers(standardized residuals within ± 3),indicating stable models.

Multiple linear regression analysis showed that the model including sex, age, energy intake, physical activity, and dinner timing significantly predicted TyG (F(5,435) = 15.19, *p* < 0.001), explaining 14.9% of the variance (R²=0.149, adjusted R²=0.139; standard error of estimate = 0.224). Among the predictors, late dinner timing emerged as the strongest independent predictor of TyG (B = 0.177, β = 0.365, t = 8.22, *p* < 0.001). The relation remained significant after adjustment for covariates, with a partial correlation of 0.367 and part correlation of 0.364. Other predictors, including sex, age, energy intake, and physical activity, were not significantly related with TyG.

Multiple linear regression showed that sex, age, energy intake, physical activity, and dinner timing significantly predicted TyG-BMI (F(5,435) = 6.77, *p* < 0.001), explaining 7.2% of the variance (R²=0.072, adjusted R²=0.062). Late dinner timing was the only significant independent predictor of TyG-BMI (B = 12.310, β = 0.265, *p* < 0.001), with significant partial (0.265) and part (0.264) correlations.

Multiple linear regression analysis showed that the model including sex, age, energy intake, physical activity, and dinner timing significantly predicted TyG-WC (F(5,435) = 13.73, *p* < 0.001), explaining 13.6% of the variance in TyG-WC (R²=0.136, adjusted R²=0.126; standard error of estimate = 58.71). Among the predictors, late dinner timing emerged as the strongest independent predictor of TyG-WC (B = 44.13, β = 0.350, t = 7.83, *p* < 0.001, 95% CI: 33.05–55.20). The relation remained significant after adjustment for covariates, with a partial correlation of 0.352 and part correlation of 0.349. Sex was also significantly related with TyG-WC (B=-14.04, β=-0.112, t=-2.49, *p* = 0.013), indicating lower TyG-WC values among females.

Multiple linear regression analysis showed that the model including sex, age, energy intake, physical activity, and dinner timing significantly predicted TyG-WHr (F(5,435) = 10.91, *p* < 0.001), explaining 11.1% of the variance in TyG-WHr (R²=0.111, adjusted R²=0.101; standard error of estimate = 0.442). Among the predictors, late dinner timing emerged as the strongest independent predictor of TyG-WHr (B = 0.288, β = 0.308, t = 6.80, *p* < 0.001, 95% CI: 0.20–0.37). The relation remained significant after adjustment for covariates, with a partial correlation of 0.310 and part correlation of 0.307. Age also showed a significant positive association with TyG-WHr (B = 0.006, β = 0.090, t = 1.97, *p* = 0.049).

Multiple linear regression analysis showed that the model including sex, age, energy intake, physical activity, and dinner timing significantly predicted TyG-WHtr (F(5,435) = 14.99, *p* < 0.001), explaining 14.7% of the variance in TyG-WHtr (R²=0.147, adjusted R²=0.137; standard error of estimate = 0.364). Among the predictors, late dinner timing emerged as the strongest independent predictor of TyG-WHtr (B = 0.268, β = 0.342, t = 7.70, *p* < 0.001). The relation remained significant after adjustment for covariates, with a partial correlation of 0.346 and part correlation of 0.341. Female sex (B = 0.104, β = 0.133, t = 2.99, *p* = 0.003) and age (B = 0.005, β = 0.093, t = 2.09, *p* = 0.037) also showed significant positive associations with TyG-WHtr.

Physical activity showed a weak inverse, although non-significant, relation with adiposity-related TyG indices (TyG-BMI, TyG-WC, and TyG-WHtr), suggesting a potential favorable metabolic influence.

**Table 8:** Multiple linear regression models assessing the relationship between TyG, modified TyG indices and dinner timings

Model	Predictor	Unstandard. Coef. B	Std. Error	Standard. Coef. Beta	t-value	*p*-value	Zero-order	Partial	Part	Tolerance	VIF
TyG	Constant	4.204	0.192	—	21.907	< 0.001	—	—	—	—	—
	Sex	0.028	0.022	0.057	1.283	0.200	0.061	0.061	0.057	0.988	1.012
	Age	0.002	0.001	0.072	1.626	0.105	0.069	0.078	0.072	0.987	1.013
	Energy intake	0.000	0.000	0.068	1.513	0.131	0.066	0.072	0.067	0.963	1.039
	Physical activity	0.000064	0.000	0.069	1.525	0.128	0.053	0.073	0.067	0.961	1.040
	Dinner timing	0.177	0.022	0.365	8.222	< 0.001	0.362	0.367	0.364	0.994	1.006
TyG-BMI	Constant	117.257	19.163	—	6.119	< 0.001	—	—	—	—	—
	Sex	1.084	2.150	0.023	0.504	0.615	0.035	0.024	0.023	0.988	1.012
	Age	-0.072	0.150	-0.022	-0.478	0.633	-0.020	-0.023	-0.022	0.987	1.013
	Energy intake	0.002	0.012	0.006	0.136	0.892	0.000	0.007	0.006	0.963	1.039
	Physical activity	-0.001	0.004	-0.012	-0.258	0.797	-0.030	-0.012	-0.012	0.961	1.040
	Dinner timing	12.310	2.150	0.265	5.725	< 0.001	0.266	0.265	0.264	0.994	1.006
TyG-WC	Constant	369.897	50.199	—	7.369	< 0.001	—	—	—	—	—
	Sex	-14.042	5.633	-0.112	-2.493	0.013	-0.102	-0.119	-0.111	0.988	1.012
	Age	0.376	0.392	0.043	0.959	0.338	0.055	0.046	0.043	0.987	1.013
	Energy intake	0.032	0.032	0.045	0.994	0.321	0.030	0.048	0.044	0.963	1.039
	Physical activity	-0.001	0.011	-0.004	-0.086	0.931	-0.012	-0.004	-0.004	0.961	1.040
	Dinner timing	44.126	5.633	0.350	7.834	< 0.001	0.346	0.352	0.349	0.994	1.006
TyG-WHr	Constant	3.566	0.378	—	9.434	< 0.001	—	—	—	—	—
	Sex	-0.078	0.042	-0.084	-1.837	0.067	-0.080	-0.088	-0.083	0.988	1.012
	Age	0.006	0.003	0.090	1.970	0.049	0.098	0.094	0.089	0.987	1.013
	Energy intake	0.000	0.000	0.052	1.133	0.258	0.039	0.054	0.051	0.963	1.039
	Physical activity	0.000032	0.000	0.018	0.383	0.702	0.012	0.018	0.017	0.961	1.040
	Dinner timing	0.288	0.042	0.308	6.795	< 0.001	0.304	0.310	0.307	0.994	1.006
TyG-WHtr	Constant	1.882	0.311	—	6.054	< 0.001	—	—	—	—	—
	Sex	0.104	0.035	0.133	2.989	0.003	0.139	0.142	0.132	0.988	1.012
	Age	0.005	0.002	0.093	2.091	0.037	0.085	0.100	0.093	0.987	1.013
	Energy intake	0.000	0.000	0.049	1.082	0.280	0.034	0.052	0.048	0.963	1.039
	Physical activity	-0.000007	0.000	-0.005	-0.106	0.916	-0.028	-0.005	-0.005	0.961	1.040
	Dinner timing	0.268	0.035	0.342	7.697	< 0.001	0.347	0.346	0.341	0.994	1.006

Footnotes: B = unstandardized regression coefficient; SE = standard error; β = standardized regression coefficient; VIF = variance inflation factor. Models were adjusted for sex, age, energy intake, physical activity, and dinner timing. Female sex was considered the reference category

## Discussion

This study primarily examined the distribution of TyG and modified TyG indices among adults with T2DM and explored their relation with dinner timing. Higher quartiles of TyG and its modified indices were characterized by less favourable anthropometric measurements, body composition, glycaemic, and lipid profiles, indicating progressive deterioration in cardiometabolic health. Participants with late dinner timing had significantly higher TyG and all modified TyG indices than early diners, with moderate effect sizes. Multiple linear regression analyses showed that late dinner timing was the strongest independent predictor of TyG and all modified TyG indices after adjustment for sex, age, energy intake, and physical activity. The zero-order, partial, and part correlations also indicated that the relation between dinner timing and TyG-derived indices persisted after accounting for the effects of the covariates. To the best of our knowledge, this is the first study to comprehensively examine the relationship between habitual dinner timing and the TyG index together with multiple anthropometric TyG derivatives among adults with type 2 diabetes mellitus.

The triglyceride-glucose (TyG) index has been increasingly applied in recent research as a surrogate marker of insulin resistance and related cardiometabolic disorders. Its widespread use is largely attributable to its simplicity and low cost, as it relies solely on fasting triglyceride and glucose measurements that are routinely available in clinical and laboratory practice [32–34]. Triglyceride-rich lipoproteins, particularly chylomicrons and very-low-density lipoproteins (VLDL), are central to this relationship. In insulin resistance, hepatic overproduction of VLDL and impaired clearance of chylomicrons contribute to elevated circulating triglyceride concentrations [35]. At the same time, hepatic insulin resistance disrupts glucose homeostasis by suppressing glycogen synthesis and enhancing gluconeogenesis, resulting in increased hepatic glucose output and higher fasting glucose levels [36]. The concurrent elevation of fasting triglycerides and glucose therefore reflects key metabolic derangements characteristic of insulin resistance, providing a physiological basis for the use of the TyG index in assessing insulin sensitivity-related metabolic abnormalities.

In the present study, visceral fat and another established insulin resistance surrogate marker TG/HDL-C ratio increased progressively across the first to fourth quartiles of TyG and its modified indices, indicating increase in cardiometabolic risk. Visceral fat accumulation is strongly associated with hepatic insulin resistance and hypertriglyceridemia, as increased portal delivery of free fatty acids and inflammatory mediators from visceral adipose tissue disrupts hepatic insulin signaling, enhances gluconeogenesis, and contributes to metabolic dysfunction. Elevated triglycerides enhance cholesteryl ester transfer protein (CETP)-mediated lipid exchange, promoting HDL catabolism, formation of small dense LDL particles, and a higher TG/HDL-C ratio [37–45]. In contrast, body muscle percentage decreased progressively from the first to the fourth quartile of TyG and its modified indices. Skeletal muscle is a major site of insulin-mediated glucose uptake and plays a central role in maintaining glucose homeostasis; therefore, reduced muscle mass may impair insulin action and contribute to greater insulin resistance, diabetes complications and cardiometabolic risk [46–49].

Human metabolism follows a circadian rhythm regulated by central and peripheral clocks. Insulin sensitivity, β-cell responsiveness, glucose tolerance, and lipid metabolism display strong diurnal variation, with metabolic efficiency highest earlier in the day and progressively declining in the evening. Insulin sensitivity declines in the evening, partly due to reduced β-cell responsiveness and decreased peripheral glucose uptake at night [50–55]. At the same time, melatonin secretion rises after dusk, promoting sleep onset but also transiently impairing insulin secretion through melatonin receptor mediated pathways in pancreatic β-cells [56, 57]. Experimental studies show that eating during periods of high melatonin levels results in higher postprandial glucose levels and reduced insulin effectiveness [58–61]. Other counter-regulatory hormones further contribute to nocturnal metabolic disadvantage. Cortisol, normally peaks in the early morning and declines through the day, irregular sleep and eating schedules may influence this circadian pattern, promoting hepatic gluconeogenesis and insulin resistance. Growth hormone, secreted in pulses during sleep, increases lipolysis and free fatty acid release, which can interfere with insulin signaling in skeletal muscle and liver [62–65]. Additionally, appetite-regulating hormones show circadian variation. Ghrelin tends to rise before meals [66] and may be dysregulated with late eating, while leptin, which normally increases at night to promote satiety and energy balance [67], may become blunted in individuals with chronic late-night food intake. When dinner is consumed late, nutrient influx coincides with a biological phase characterized by reduced insulin efficiency, increased lipolytic activity, and altered appetite hormone signaling. This hormonal environment favors higher nocturnal glucose levels, impaired triglyceride clearance, and greater hepatic lipid synthesis. Over time, these effects may elevate fasting glucose and triglyceride concentrations, thereby increasing TyG and modified TyG indices. Consuming a large proportion of daily energy intake late at night may therefore lead to exaggerated postprandial glucose excursions, impaired triglyceride clearance, and increased hepatic de novo lipogenesis. Over time, these alterations may elevate fasting glucose and triglyceride concentrations.

Peripheral clocks in the liver, pancreas, adipose tissue, and skeletal muscle are entrained not only by light but also by feeding timing. Late-night eating may desynchronize these peripheral clocks from the central circadian pacemaker, leading to circadian misalignment. This misalignment may impair insulin signaling pathways, reduce glucose uptake in skeletal muscle, and increase hepatic glucose output [51, 59, 68–70]. Furthermore, nocturnal elevations in triglycerides may reflect reduced activity of lipoprotein lipase and altered lipid oxidation rhythms, further amplifying TyG values [71]. The stronger associations observed with modified TyG indices that incorporate central obesity measures suggest that circadian disruption may particularly exacerbate visceral fat related metabolic dysfunction. Visceral adipose tissue is metabolically active and sensitive to hormonal and circadian influences, promoting increased free fatty acid flux to the liver during periods of metabolic inefficiency, such as late evening hours [72–75].

Intrinsic circadian clocks within adipocytes play a key role in regulating critical adipose tissue functions, including lipolysis, adipocyte differentiation, inflammatory signaling, browning, thermogenic activity, and the rhythmic production and release of adipokines [76]. Disruption of these local clocks can therefore modify adipose tissue physiology and compromise whole-body energy balance. Both experimental and clinical studies have showed rhythmic expression of clock-related genes across multiple white adipose tissue depots in rodents [74, 77, 78] as well as in humans [79–81], using in vitro and in vivo approaches. 

This study has several limitations that should be considered when interpreting the findings. First, its cross-sectional design precludes inference of causality between dinner timing and insulin resistance related markers. Second, visceral fat was assessed using a bioelectrical impedance analysis (BIA)-derived estimate rather than a gold-standard imaging technique. Additionally, the study population comprised adults with T2DM in a specific geographic and ethnic context, which may limit the generalizability of the findings to other populations. Future longitudinal and interventional studies are warranted to clarify causality and determine whether habitual early dinner timing, independent of total calorie intake, influences insulin resistance and contributes to reducing adverse cardiometabolic risk.

## Conclusions

This study shows that later dinner timing is significantly related with higher surrogate insulin resistance markers TyG and modified TyG indices among adults with type 2 diabetes mellitus. These findings support emerging evidence that meal timing, independent of total energy intake, may influence insulin resistance through circadian misalignment, hormonal fluctuations, and impaired nocturnal glucose and lipid metabolism.

TyG and modified TyG indices were significantly related with lower muscle percentage, higher visceral fat percentage, reduced HDL-C concentrations, and elevated adverse lipemic and glycaemic parameters. The original TyG index showed stronger differentiation of biochemical markers, whereas the modified TyG indices showed stronger differentiation across their corresponding anthropometric components, along with stronger differences in body composition and selected biochemical parameters. These findings suggest that modified TyG indices may provide complementary information on adiposity and metabolic risk beyond the original TyG index in individuals with type 2 diabetes mellitus.

Given the observed relation of late dinner timing with higher TyG and modified TyG indices, incorporating chrononutrition considerations into diabetes management, alongside conventional dietary strategies, may contribute to reducing the risk of diabetes-related complications in individuals with type 2 diabetes mellitus.

## Data Availability

The datasets generated during the current study will be made available by the corresponding author upon reasonable request.

## Abbreviations

BMI: Body Mass Index
HDL-C: High–density lipoprotein cholesterol
HbA1c: Glycated haemoglobin/Glycosylated hemoglobin
LDL-C: Low–density lipoprotein cholesterol
MET-min/week: Metabolic equivalent minutes per week
T2DM: Type 2 diabetes mellitus
TG: Triglycerides
TG/HDL-C ratio: Triglyceride to high–density lipoprotein cholesterol ratio
TyG: Triglyceride–glucose index
TyG-BMI: Triglyceride–glucose index Body Mass Index
TyG-WC: Triglyceride–glucose index waist circumference
TyG-WHr: Triglyceride–glucose index waist–to–hip ratio
TyG-WHtr: Triglyceride–glucose index waist–to–height ratio
WC: Waist circumference
WHr: Waist–to–hip ratio
WHtr: Waist–to–height ratio
